# Repeat hospital transfers among long stay nursing home residents: a mixed methods analysis of age, race, code status and clinical complexity

**DOI:** 10.1186/s12913-022-08036-9

**Published:** 2022-05-10

**Authors:** Amy Vogelsmeier, Lori Popejoy, Elizabeth Fritz, Kelli Canada, Bin Ge, Lea Brandt, Marilyn Rantz

**Affiliations:** 1 Sinclair School of Nursing, University of Missouri, Columbia, MO USA; 2 School of Social Work, University of Missouri, Columbia, MO USA; 3 School of Medicine, University of Missouri, Columbia, MO USA

**Keywords:** Nursing homes, Nursing home residents, Hospital transfers, Advanced practice registered nurses

## Abstract

**Background:**

Nursing home residents are at increased risk for hospital transfers resulting in emergency department visits, observation stays, and hospital admissions; transfers that can also result in adverse resident outcomes. Many nursing home to hospital transfers are potentially avoidable. Residents who experience repeat transfers are particularly vulnerable to adverse outcomes, yet characteristics of nursing home residents who experience repeat transfers are poorly understood. Understanding these characteristics more fully will help identify appropriate intervention efforts needed to reduce repeat transfers.

**Methods:**

This is a mixed-methods study using hospital transfer data, collected between 2017 and 2019, from long-stay nursing home residents residing in 16 Midwestern nursing homes who transferred four or more times within a 12-month timeframe. Data were obtained from an acute care transfer tool used in the Missouri Quality Initiative containing closed- and open-ended questions regarding hospital transfers. The Missouri Quality Initiative was a Centers for Medicare and Medicaid demonstration project focused on reducing avoidable hospital transfers for long stay nursing home residents. The purpose of the analysis presented here is to describe characteristics of residents from that project who experienced repeat transfers including resident age, race, and code status. Clinical, resident/family, and organizational factors that influenced transfers were also described.

**Results:**

Findings indicate that younger residents (less than 65 years of age), those who were full-code status, and those who were Black were statistically more likely to experience repeat transfers. Clinical complexity, resident/family requests to transfer, and lack of nursing home resources to manage complex clinical conditions underlie repeat transfers, many of which were considered potentially avoidable.

**Conclusions:**

Improved nursing home resources are needed to manage complex conditions in the NH and to help residents and families set realistic goals of care and plan for end of life thus reducing potentially avoidable transfers.

## Introduction

Nursing home (NH) residents are at increased risk for hospital transfers resulting in emergency department (ED) visits, observation stays, and hospital admissions [1–3]. Hospital transfers can also result in adverse outcomes e.g., hospital-acquired infections as well as increased costs [4]. According to a report by the Office of Inspector General, NHs transferred approximately 25% of their residents for hospital admission, at a cost to Medicare of $14.3 billion [5]. Studies suggest between 33 to 66% of NH residents return to the hospital at least once within 12 months [6–8], and 11% to 18% experience frequent or repeat transfers, defined by some researchers as four or more transfers within a year [7, 8]. Evidence suggests between 20 to 67% of NH to hospital transfers may be avoidable [1, 2].

NH residents who transfer are typically younger in age, have multiple chronic conditions e.g., advanced dementia, congestive heart failure, chronic obstructive pulmonary disease, and complex care needs [8–12]. Factors associated with potentially avoidable hospital transfers include resident and family influences such as requests to transfer and/or lack of adequate advance care planning [13, 14]. Organizational influences underlying potentially avoidable transfers include limited NH resources to manage ill residents in the NH and lack of staff knowledge about how to assess and manage complex conditions [2, 10, 15].

Despite what is known about hospital transfers overall, less is known about residents who experience repeat transfers. Grunier and colleagues [7] found residents with four or more ED transfers in a year were often younger than 75 years old, more likely to be male, and less likely to have Alzheimer’s disease or other related disorders. It is important to consider what unique demographic and transfer characteristics exist for residents with repeat transfers that may have contributed to their hospital transfer. Understanding these characteristics more fully will help identify appropriate intervention efforts needed to reduce repeat transfers.

Given that existing evidence about residents who experience repeat transfers is scant, there are two aims for this mixed-methods analysis. Aim one is to describe characteristics of age, race, and code status (do-not-resuscitate [DNR], full code, or mixed [resident’s code status changed between DNR and full code]) for residents with repeat transfers, defined for this study as four or more transfers within a 12-month timeframe. Aim two is to describe the clinical, resident/family, and organizational influences contributing to repeat transfers among select residents who had eight or more transfers during the study timeframe.

## Methods

### Design

This was a retrospective explanatory mixed-methods design. We first analyzed quantitative data, then analyzed qualitative data based on the quantitative findings [16]. Data for this analysis were collected as part of the Missouri Quality Initiative (MOQI), Centers for Medicare and Medicaid (CMS) Innovations Center demonstration project implemented over eight years (2012–2020) and included two phases [17]. Phase One of the MOQI intervention included advanced practice registered nurses (APRNs) embedded full-time in 16 NHs in and around a large urban area in Missouri with the goal to reduce avoidable hospitalizations for long-stay NH residents. In 2016, Phase Two began with some refocusing, but the goal of reducing avoidable hospitalizations continued [18–20]. The 16 MOQI NHs were a mix of urban, suburban, and rural, ranged in size from 120 to over 300 beds, and were predominantly for-profit (*n*= 14, 88%). The total sample of residents enrolled in MOQI were dual eligible for Medicare and Medicaid services. The enrollment numbers averaged 1819 residents in 2014 to 1068 in 2019; the declining enrollment is explained in other publications[18–20]. We chose 2017 to 2019 for the repeat transfer analysis because additional data regarding transfers were collected during Phase Two, enabling a more detailed analysis. The total sample of residents enrolled in MOQI during these three years averaged 1482 residents in 2017, 1210 in 2018, and 1068 in 2019 [19]. Short-stay residents defined as those residing in the NH less than 100 days or residents receiving hospice services were excluded from the demonstration project.

### Data

As part of the MOQI project, APRNs documented retrospective details about every resident’s hospital transfer into an adapted INTERACT acute care transfer (ACT) tool using Qualtrics survey software [21, 22]. The ACT tool contains open- and closed-ended questions about each acute care transfer. Minimal adaptations included adding resident race and specifics about the APRN and RN role in hospital transfer decisions. Data from the INTERACT ACT tool for the 113 residents were downloaded from the Qualtrics database into an Excel file for this analysis.

Because the INTERACT tool captures information about individual transfers, we first organized the data according to the resident’s unique identifier in the parent study to link each resident to their transfers occurring during the 2017–2019 study timeframe. Age, race, and code status, abstracted from the resident’s medical record and recorded by the APRN in the ACT tool for each transfer, were then analyzed as resident-level variables. Although race was a constant variable recorded as part of each resident’s transfer, age and code status were not; residents continued to age over time and code status varied for some residents at the time of transfer. We used the resident’s date of birth to calculate their age as of 2017 (start of this study timeframe) and to account for variation in code status, we assigned one of three categories to each resident: persistent DNR, persistent full code, and mixed. *Persistent DNR* and *Persistent Full Code* were assigned when all, or all but one transfer was coded the same. For example, a resident who had 10 transfers with nine transfers recorded as full-code and one as DNR was assigned as *Persistent Full Code*. Resident’s code status was assigned as *Mixed* when code status at transfer varied two or more times. For example, a resident who had eight transfers with five recorded as full-code and three as DNR was assigned as *Mixed*.

Data for the qualitative analysis included select open- and closed-responses from the ACT tool. Open-text responses included APRN documentation about the change that led to the transfer, how changes were evaluated and managed prior to the transfer, and what changes in care processes are needed. Closed-ended responses included major diagnoses at admission, diagnosis/presumed diagnosis at transfer, and other factors (e.g., resident/family request, resources available/unavailable to manage within the NH, APRN consulted and/or examined the resident prior to transfer, missed early illness signs, staff experience/comfort with clinical care). Data also included as part of the ACT tool completion was APRN determination about whether a transfer was deemed potentially avoidable (yes/no). The APRN and MOQI RN supervisor made the final determination about potential avoidabilty after each transfer using chart review and nurse interviews to identify root cause(s) of why the transfer occurred [22].

### Sample

**Fig. 1:**
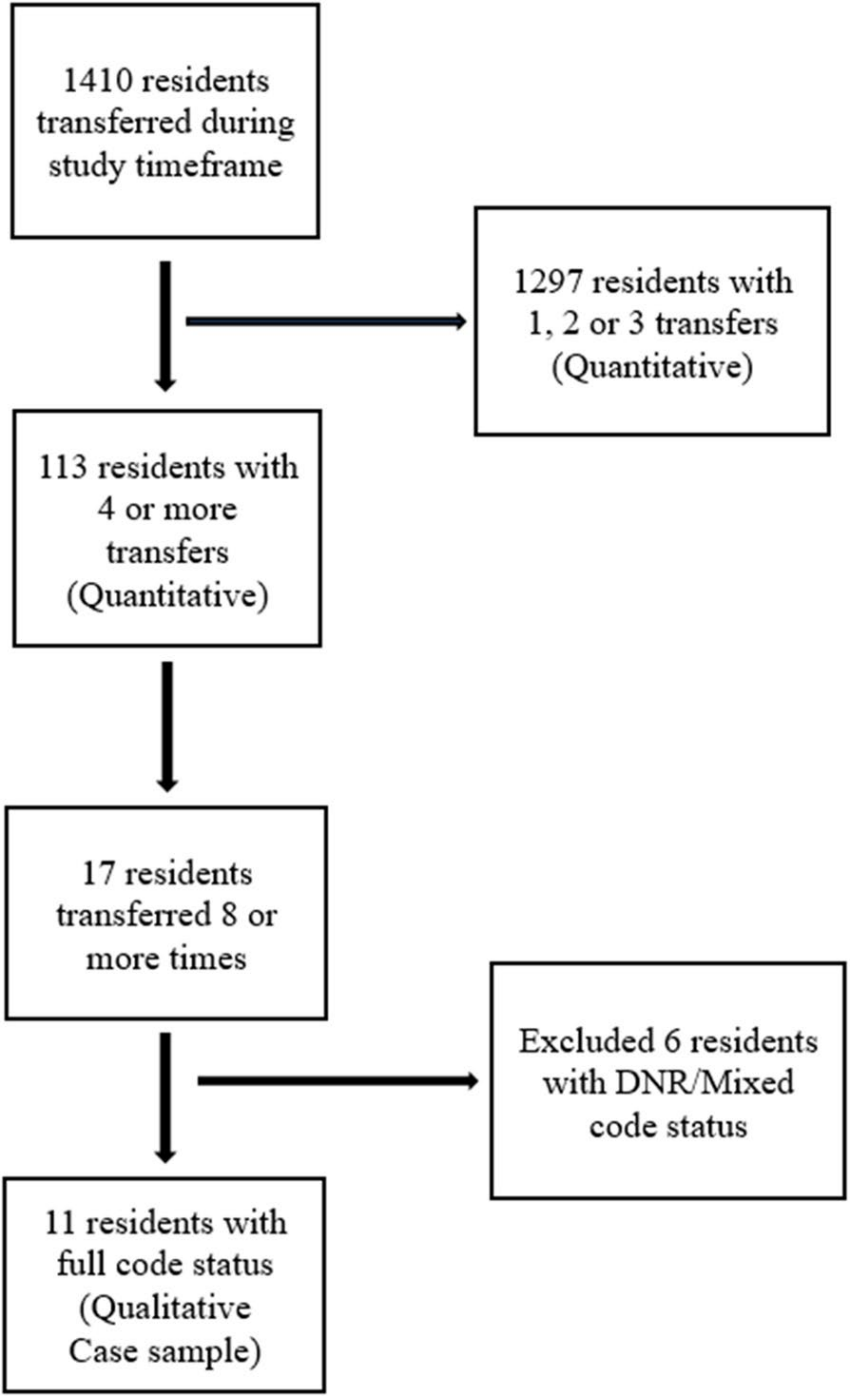
Sample selection flowchart

Among the MOQI residents enrolled between 2017 and 2019, 1410 residents had at least one hospital transfer. For the quantitative sample, we identified 113 residents (8%) who were transferred four or more times within a 12-month timeframe. The 113 residents had a total of 609 transfers during the three-year observation. Of these 113, 17 were transferred eight or more times; 11 of the 17 were identified as full-code, 5 were DNR, and one was mixed. The sample of residents selected for the qualitative analysis included those residents with eight or more transfers and identified as full-code status (*n *= 11). The deliberative sample of residents with eight or more transfers and full-code status was selected to offer the richest data set possible so patterns could be identified for each resident’s set of repeat transfers without the confounding variation of DNR or mixed [23]. In total, these 11 residents had 121 transfers throughout the three-years. See Fig. 1 Sample selection flowchart.

### Data analysis

For the quantitative analysis, we used the chi-square test to assess group differences based on resident age, race, and code status. First, we compared differences in age and race of the 113 residents who had repeat transfers to the 1297 MOQI residents who did not. Second, we compared residents within the repeat transfers group to identify differences based on code status and race also using a chi-square test. A *p *-value less than 0.05 was considered statistically significant. All analyses were done using SAS 9.4 software (SAS Institute Inc. Cary, NC).

For the qualitative analysis, Qualtrics data for the 11 residents’ transfers were downloaded into an excel database for coding. Hsieh and Shannon’s [24] procedure for directed content analysis was used whereby a priori categories were developed to guide the coding. Four PhD-prepared researchers and a doctoral student discussed contributors to hospital transfers based on existing literature and agreed on four a priori categories: 1) clinical condition, 2) residents/family, 3) organizational factors, and 4) goals of care discussions. For each resident, data were coded by reviewing the transfer data line by line. Initially, text from three residents’ transfers (*n* = 34 transfers) were coded jointly by the team to establish consistency. This process involved all members reading through the transfer data together, identifying salient texts and assigning representative codes. The remaining eight residents’ transfers (*n* = 87 transfers) were coded by a single team member (AV) with a second member (EF) independently reviewing codes for three of those eight residents’ transfers (*n* = 35 transfers) to confirm consistency. Once initial codes were assigned, they were reviewed and organized into sub-categories under each of the four a priori categories. During the next phase of analysis, the sub-categories were merged when there was overlap between the categories. The a priori category of “goals of care discussion” was collapsed with “resident/family” because text about goals of care discussions co-occurred within the resident/family category. The findings were reviewed and agreed upon by the team as presented below under the section Qualitative findings.

## Results

### Quantitative findings

**Fig. 2:**
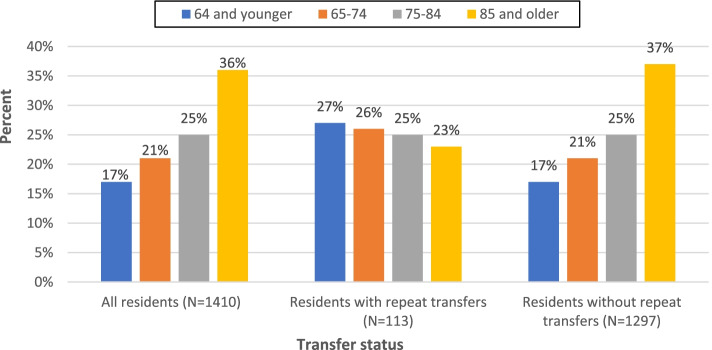
Distribution of all transfers by age. * *p* < .05; Repeat transfers = 4 or more per year

**Fig. 3:**
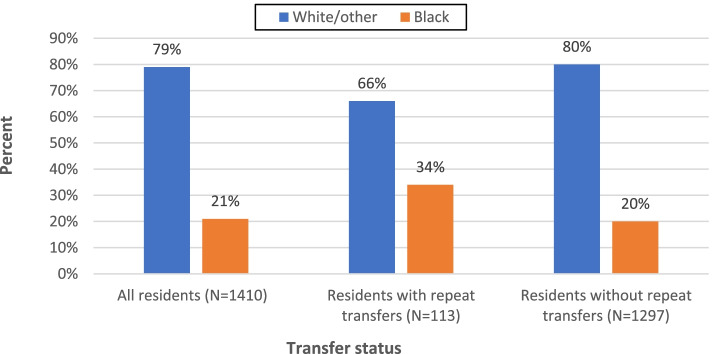
Distribution of all transfers by race. * *p* < .001; Repeat transfers = 4 or more per year

Residents with four or more transfers per year had a mean age of 72 years and were primarily White (*n* = 75, 66%) and Black (*n* = 38, 32%); less than one percent were Asian, Hispanic/Latinx, or other/multiracial. When analyzing for differences in age, residents in the repeat transfers group were significantly younger than those without repeat transfers (*p* = 0.004) and were significantly more likely to be Black (34% vs 20%, *p* < 0.001). Figures 2 and 3 depict the distribution of all transfers by age and race, respectively.

**Fig. 4:**
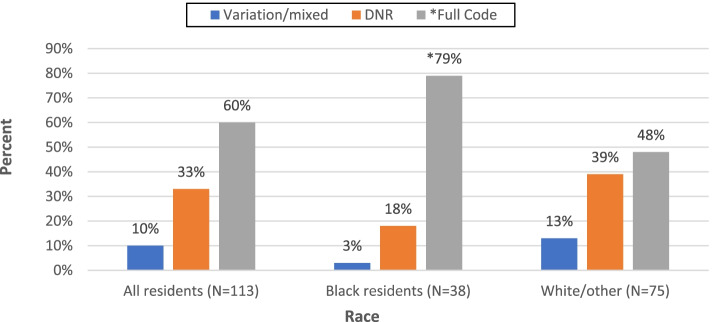
Distribution of repeat transfers by code status and race. *p < .05; Repeat transfers = 4 or more per year

When comparing code status within the repeat transfers group, 60% (*n* = 66) were full code, 33% (*n* = 36) were DNR and 10% (*n* = 11) were mixed. Black residents were significantly more likely to be full code (*n* = 30, 79%; *p* < *0.05*) when compared to White residents. Figure 4 depicts the percent distribution of repeat transfers by both code status and race.

### Qualitative findings

#### Sample description

Out of the 113 residents with repeat transfers, 11 (10%) met the criteria for 8 or more transfers and Persistent Full-Code status. These 11 residents averaged 11 transfers each (range 8 to 15) and accounted for 20% (*n* = 121) of the total transfers (*n* = 605) among all residents with repeat transfers. Most residents with eight or more transfers were Black (*n* = 6; 55%), female (*n* = 6, 55%), and the vast majority were 64 years of age or younger (*n* = 10; 91%) with an average age of 55 years (range 38 to 67). Ten of the 11 residents were initially admitted to the NH with chronic medical conditions and/or mental illness diagnoses; one was admitted due to an anoxic brain injury. Overall, 74% of transfers resulted in hospitalization (*n* = 90 transfers). Only two residents whose repeat transfers were fall-related were predominantly ED visits only. The majority of transfers for the 11 residents were deemed by the APRNs to be potentially avoidable (*n* = 64; 53%). Summary data for the 11 residents and their transfers are presented in Table 1.

**Table 1:** Summary of Resident Transfer Data

ID	Age (in yrs) Race/Sex # Transfers (%) Denotes percent potentially avoidable	Signs/symptoms and/or diagnoses at transfer (#) Denotes number of times this condition/diagnosis was present at transfer	Clinical needs	Medical/ Mental health history	Resident/Family Request to transfer	Transfer Outcome/
#1	61 years White female 8 transfers (86%)	Fall with head injury (i.e., laceration, bleeding) (5), altered mental status (2), unresponsiveness (2), fall with other injury (1), seizure (1), wounds (1)	None reported	Seizures Chronic pain Depression Anxiety	None reported	Admit (1) ED (7)
#2	53 years Black male 9 transfers (33%)	Elevated temperature (6), Sepsis (5), UTI (2), wound infection (2), fall (1), hematuria (1), acute renal failure (1), colostomy (planned) (1)	Wound care Colostomy Central line Foley catheter	Diabetes Multiple Sclerosis Depression	None reported	Admit (8) Missing (1)
#3	67 years White female 9 transfers (44%)	Shortness of breath (4), GI bleeding (2), bleeding, other (2), emergent dialysis (1), low pulse oximetry (1)	Dialysis Wound care	ESRD COPD	Resident (2)	Admit (9)
#4	49 years White female 10 transfers (40%)	Shortness of breath (3), wound infections (3), altered mental status (2), COPD (1), sepsis (1), respiratory failure (1), hypoglycemia (1), unresponsive (1), low pulse oximetry (3), elevated temperature (1)	Dialysis Wound care Supplemental Oxygen	ESRD COPD CHF Seizure Depression Anxiety	None reported	Admit (9) Missing (1)
#5	58 years Black male 10 transfers (30%)	Hyperglycemia (4), hypoglycemia (2), emergent dialysis (1), dialysis shunt malfunction (1), shortness of breath (1), unresponsiveness (1), low hemoglobin (1), altered mental status (1), pneumonia (1), UTI (1)	Dialysis	ESRD DKA	Resident (1)	Admit (8) ED (1) Obs (1)
#6	64 years Black female 10 transfers (70%)	Fall w/ injury to head (9), fall w/ other injury (1), UTI (1)	None reported	COPD Parkinson’s Alcoholism	None reported	Admit (3) ED (7)
#7	62 years Black male 11 transfers (27%)	GI bleeding (7), UTI (5), pneumonia (2), sepsis (2), hematuria (1), seizure (1), G-tube malfunction (1), shortness of breath (1), elevated temperature (1), low pulse oximetry (2)	Tracheostomy Foley catheter G-tube Oxygen	CVA Seizures Anxiety	None reported	Admit (9) ED (2)
#8	64 years White female 11 transfers (55%)	UTI (7), kidney stone removal, (4), sepsis (2), elevated temperature (1)	None reported	Diabetes Chronic pain Depression	None reported	Admit (10) Obs (1)
#9	44 years Black male 14 transfers (50%)	Sepsis (7), UTI (8), hematuria (6), pneumonia (3), respiratory failure (3), GI bleeding (1), shortness of breath (3), low pulse oximetry (3), elevated temperature (1)	Tracheostomy Foley catheter G-tube Oxygen	Anoxic brain injury Anxiety	Family (10)	Admit (12) ED (1) Obs (1)
#10	45 years Black male 14 transfers (86%)	Seizures (10), UTI (5), pneumonia (2) hematuria (1), shortness of breath (2); elevated temperature (1), low pulse oximetry (1)	Suprapubic catheter G-tube	Seizures Multiple Sclerosis Schizophrenia	Family (11)	Admit (9) ED (3) Missing (2)
#11	38 years White female 15 transfers (53%)	Shortness of breath (9), pneumonia (4), COPD (3), acute psychiatric care (2), tracheostomy tube replaced (2); elevated temperature (1)	Tracheostomy Oxygen	COPD Depression Anxiety Bipolar disorder	Resident (7)	Admit (12) ED (3)

*UTI* Urinary tract infection, *ESRD* end stage renal disease, *DKA* diabetic ketoacidosis, *DM* diabetes mellitus, *COPD* chronic obstructive pulmonary disease, *CVA* cerebral vascular accident, *G-tube* gastrostomy feeding tube

*Admit* Hospital admission, *ED* Emergency Department visit only, *Obs* 24-h hospital observation stay, *Missing* transfer outcome not reported

#### Transfer characteristics

To better understand salient features and contributors to repeat transfers, researchers examined the 121 transfers that occurred across the 11 residents. The clinical condition, resident/family influence, and organizational factors for each transfer were explored and relevant themes and patterns are discussed below and summarized in Table 2.

**Table 2:** Summary of Themes for 8 or more cases

Residents’ Clinical Condition	Resident/Family Influences	NH Organizational Factors
Recurring acute diagnoses including sepsis, pneumonia, urinary tract infections, GI bleed, respiratory failure, DKA	Requests for transfers due to concerns about new clinical problems or exacerbations of chronic problems	Lack of NH resources to manage acute conditions such as need for suturing, management of acute illness
Chronic medical conditions including history of seizures, chronic pain, diabetes, ESRD, CHF, COPD, multiple sclerosis, Parkinson’s, stroke, anoxic brain injury	Missed opportunities between residents/families and staff for goals of care discussions and advance care planning	NH resources available, not accessed by staff such as APRNs not consulted prior to transfer
Repeat falls		
Mental health history such as anxiety, depression, alcoholism, bipolar disorder		Lack of staff experience or comfort providing complex care such as managing tracheostomy tubes, feeding tubes, complex wounds
Chronic care needs including tracheostomies, wound care, colostomies, dialysis, central lines, foley catheters, suprapubic catheters, gastric feeding tubes		Missed nursing care such as missed assessment, not obtaining specimens, not obtaining vital signs

*GI* Gastrointestinal, *DKA* Diabetic ketoacidosis, *ESRD* End stage renal disease, *CHF* Congestive heart failure, *COPD* Chronic obstructive pulmonary disease

### Clinical condition

Documentation about the residents’ clinical conditions represented both their underlying chronic illnesses and the complex care needs required for those conditions. Most transfers involved exacerbations of the resident’s chronic underlying conditions; many deemed unavoidable based on resident acuity at the time of transfer. For example, Residents #3, #4, and #11 were each transferred multiple times for respiratory signs/symptoms (i.e., “shortness of breath,” “low pulse oximetry”) resulting from COPD exacerbations, repeat pneumonia, and/or “respiratory distress.” In another example, Resident #5, who had a history of diabetic ketoacidosis (DKA) had six out of 10 transfers related to “hyperglycemia” (e.g., blood glucose “ > 500” [mg/dl]; “ > 900” [mg/dl]) or hypoglycemia (e.g., blood glucose “53” [mg/dl]) with the remaining transfers related to end stage renal disease. Resident #7 had a “G-tube” [gastrostomy feeding tube], Foley catheter, and tracheostomy and had repeat transfers for “GI [gastrointestinal] bleeding” and/or healthcare associated infections such as “UTIs” [urinary tract infections] or pneumonia.

Many residents had complex nursing care needs related to their treatments required for chronic conditions including tracheostomies, long-term urinary catheters, suprapubic catheters, gastric feeding tubes, supplemental oxygen, wound care, and/or dialysis. Eight residents also had histories of mental health diagnoses including depression, anxiety, schizophrenia, or bipolar disorder.

### Resident/family influences

APRN documentation about resident and family influences related most frequently to requests for transfers and to a lesser extent about the need to explicate goals of care and preferences for care to avoid the transfer. Nearly half of the residents requested or had family request transfers at different points in time. For example, Resident #3 requested to be transferred twice, once with “increased bleeding from foot wound, unable to stop, and resident requested to go to hospital” and again, after they “refused dialysis…and then c/o [complained of] not feeling well and requesting to go to the ER.” Another example included APRN documentation about Resident #4 that stated, “BP [blood pressure] elevated, new order to increase hydralazine…then with altered mental status, SOB [shortness of breath], and [resident] requesting hospitalization.”

Resident and family requests to transfer were identified as a pattern for three of the residents (#9, #10, #11) who were also the youngest residents in this sample. Resident #11, who was 39 years old, requested to transfer seven out of 12 transfers. This person had frequent infections, chronic illness exacerbations, a tracheostomy tube, and mental health diagnoses of depression, anxiety, and bipolar disorder. APRN documentation indicated this resident would frequently “request” and/or “insist” on going to the ED/hospital for treatment. In one instance, the APRN documented, “CN [charge nurse] explained the hospital is not going to do anything we are not doing now.”

Both residents #9 and #10 had frequent transfer requests by family members. Resident #9, a 44-year-old with a frequent infections, bleeding, and care needs including a tracheostomy, Foley catheter, gastric tube feeding, and supplemental oxygen along with a history of anxiety, had family requests to be “sent to the hospital” for 10 of 14 transfers. Resident #10 was 45 years old, had frequent seizures and infections, a history of schizophrenia and had family request to be transferred for 11 out of 14 transfers. APRN documentation for both residents included phrases such as, “transfer per family request,” “DPOA (Durable Power of Attorney) request resident be sent to ER (emergency room)” or “Wife requested be sent out for evaluation.” APRNs documented the need for having goals of care and care preference discussions with two of the residents requesting transfers (#10, #11) noting in retrospect that the absence of these discussions may have contributed to potentially avoidable transfers.

### Organizational factors

Organizational factors contributing to repeat transfers included references by the APRNs about NH resources, including lack of resources and resources not accessed, lack of staff expertise or comfort in providing care, and missed nursing care that may have contributed to the resident’s change in condition. NH resources most often related to resources not being available to manage the resident in the NH. For example, Residents #1 and #6 had multiple falls requiring laceration repairs that the NH could not provide. Similarly, Resident #7 had frequent GI bleeding often requiring emergent care including blood transfusions. Additionally, five residents (# 2,4,7,8,9) were transferred for sepsis requiring intravenous antibiotics and fluids and close monitoring, with care needs exceeding what the NH could provide. The majority of these transfers were often deemed by APRNs to be unavoidable due to resident acuity at the time of transfer and the need for a higher level of care.

APRNs noted potentially avoidable transfers often occurred when NH resources were available but not accessed prior to transfer. For example, Resident #7 transferred for a malfunctioning gastric tube and later a malfunctioning suprapubic catheter, and Resident #11 had problems with a tracheostomy tube; all were transferred despite having onsite resources to manage and replace the tubes. Other transfers occurred without prior management attempts such as Resident #5 who transferred for low blood sugars and Resident #10 for seizures despite having medications to initially treat these conditions inhouse.

APRNs documented attempts were made by nursing staff to manage residents in the NH for some transfers, often with the assistance of the APRN, but subsequently changes in their condition required transfer to a higher level of care, resulting in unavoidable transfers. For example, when Resident #11 began experiencing respiratory symptoms that worsened over time despite the APRN initiating breathing treatments and oxygen days prior to the transfer. At other times, APRNs were notified after transfer orders were in place or when emergent conditions occurred, such as when the APRN was called to examine Resident #9 who was, “noted to have dyspnea with increased heart rate and fever. Oxygen saturation 90–92% on 6 [liters] oxygen.” However, most often, transfers across the 11 residents occurred without APRN involvement prior to transfer thus resulting in transfers that might have been avoided. Reasons included the APRN not being on site (e.g., transfers occurring on weekends, evenings, nights) and/or not being notified by staff despite being available (e.g., APRN working on a different unit, available on call).

Missed nursing care, an underlying factor associated with many potentially avoidable transfers, was evident across residents’ transfers and included documentation about “assessment not completed,” “VS (vital signs) not documented,” “monitoring” not occurring, missed “early illness signs” and specimens “ordered, but not collected.” In addition, APRNs consistently documented concerns about lack of staff experience and/or comfort with complex residents. For example, staff inexperience to manage Residents #4 and #5 who experienced both hyper/hypoglycemia or Resident #10 who had frequent seizures. Additionally, staff lack of experience/comfort with complex clinical needs were documented for Resident #2, #3, and #4 who had wound care and other clinical needs or Residents #7, #9, and #11 who each had tracheostomies and other indwelling lines.

## Discussion

This study adds new findings to what is known about NH transfers. While other research has revealed similar evidence that residents of younger age, full code, and clinically complex are more likely to be transferred [9–11]; few studies have examined repeat transfers. When considering this unique population, we found residents with repeat transfers were significantly younger when compared to the larger MOQI population, and similar to those reported in a study by Blackburn et al. [9] Younger resident admissions to NHs typically occur either as a result of trauma or debilitating events such as stroke, have complex multimorbidities with aggressive management such as G-tubes, tracheostomies and are thus more complex to manage [25]. These complex conditions, particularly in younger residents, require goals of care conversations and advance care planning that includes realistic treatment options and discussions about code status; however, families of younger residents may not be as emotionally prepared to have these conversations. Typically, younger NH residents, even those with complex medical conditions that equate to high mortality risk, tend to remain full code as compared to older adults with similar clinical presentations [26]. In the qualitative cases presented in this study, the residents were all younger and remained full code after having been admitted to the NH following a catastrophic injury or severe chronic illness. While the tendency for residents/families may be to choose full code over DNR status, the reality of 30-day survival following cardiopulmonary resuscitation (CPR) in NHs is less than 2% [27]. Possible reasons for the reluctance toward DNR may be that residents, families and staff conflate code status with treatment options assuming DNR means no treatment at all which is not the case. DNR simply means there is an order for a medical intervention to attempt resuscitation after cardiac/pulmonary arrest. Therefore, having realistic goals of care conversations that include treatment decisions and advance care planning along with an informed code status determination become vital tools to support residents and families.

NH residents with advance care planning are less likely to experience avoidable hospitalizations [28]. Anecdotally, APRNs discussed challenges associated with managing younger residents who are repeatedly transferred to the hospital, including the perceived reluctance of families to have goals of care conversations that would include treatment options and end of life care. Studies suggest factors such as co-morbid conditions and physical dependence which are reflected in our 11 cases are associated with higher mortality [29, 30]. Although not clear from our data, it is possible that NH staff (e.g., nurses, social workers) missed opportunities to have goals of care conversations or perhaps they attempted discussions but were met with reluctance from family or residents. Anecdotally, MOQI APRNs noted the importance of having ongoing, thoughtful discussions with residents and families about treatment and realistic goals of care.

In addition to age, our findings suggest Black residents are significantly more likely to experience repeat transfers. In the US, Black residents comprise 14% of the NH population [31], yet in our study they accounted for 20% of all transfers and 32% of repeat transfers. Black residents are more likely to be disabled on admission, have complex care needs, reside in low quality NHs, and receive sub-standard care [32–35], all factors placing them at higher risk for transfers. In general, goals of care conversations are less likely to occur among Black individuals than among those who are white [36, 37]. Perhaps this disparity is in part because NH staff assume Black residents and their families are reluctant to engage in goals of care conversations and advance care planning in favor of more aggressive treatment [38]. It is important to note that longstanding structural racism likely contributes to poor health outcomes both before being admitted to the NH as well as during their NH stay [39]. It is also possible that lack of trust of the healthcare system among Black residents and families is a result of structural racism.

Although repeat transfer data from this project do not provide detail to explain the race disparity, the assertion about race and NH quality does not seem to fit. While MOQI NHs had varying quality over time, the majority reduced avoidable hospital transfers [19], improved quality measure scores [20, 40], and enhanced staff workflow [41] suggesting that, in this study, reasons for higher transfers among Black residents were unlikely related to poor NH quality.

Our findings that organizational influences such as limited NH resources and limited staff capacity are contributors to hospital transfers also confirm existing evidence about characteristics of potentially avoidable NH resident transfers [10, 15]. However, patterns noted in this study related to chronic illness exacerbations and ongoing complex care needs highlight missed opportunities to assure resources were in place to manage these anticipated events. Moreover, our finding about missed nursing care, defined as required nursing care including documentation of care that is either omitted or delayed [42, 43] is concerning yet consistent with other studies [44–46]. For example, White and colleagues [45] found 72% of NH registered nurses reported missing one or more necessary care tasks including resident surveillance, patient/family teaching, and care planning, factors which likely contribute to the need for hospital transfers [46].

Comments by APRNs about staff discomfort or inexperience with complex conditions and equipment suggest a need for better staff development to prevent unnecessary transfers. This is consistent with findings by Cooper et al [47] that nurses are inadequately prepared for work in NHs, specifically requiring more training in managing chronic conditions. Better training and staff development may help reduce the frequency of missed nursing care and lead to earlier assessment and intervention.

Perhaps most evident is that APRNs were not accessed by staff prior to many of the repeat transfers, either due to unavailability or not being notified by staff at the time of condition change. Based on findings from the MOQI parent study, APRNs, when consulted, were successful in reducing potentially avoidable hospital transfers for the majority of MOQI NHs [19, 20]. Additionally, in MOQI the APRNs received coaching and ongoing support on having goals of care and end of life discussions. As a result, APRNs, coupled with social work support were effective in having goals of care and advance care planning discussions which also influenced resident outcomes [48]. This may not be the case in many NHs where resources may not include APRNs, licensed social workers, and adequate numbers of registered nurses to manage clinically complex residents or to have goals of care and end of life discussions with families [49]. More work is needed at the national level to assure necessary NH resources are accessible including APRNs, registered nurses, and licensed social workers to close the gap in providing complex clinical care and end of life communication between NH staff and residents.

### Practice implications

Based on findings from this study, NH residents with repeat transfers were younger and largely were full code. These findings coupled with the overrepresentation of Black residents in the repeat transfer group suggest a need for both evidenced-based and culturally appropriate strategies for goals of care discussions and, more generally, different approaches to advance care planning. APRNs, registered nurses, and clinical social workers are key NH staff needed to engage residents and families in difficult conversations around resident change in status and end of life care planning [48–50]. Resident and family engagement in difficult conversations is critical to build rapport, trust, and understanding of the NH’s capacity to address the needs of the residents. For family members, staff acknowledging and respecting their role as resident advocates is an important part of building their trust in the NH staff to manage complex needs. These professionals are trained to use effective communication strategies and approaches that take into consideration a resident and family’s cultural background [51, 52]. Finally, NH leaders and staff need to reflect on their assumptions about residents and families so that long standing biases and racial tendencies are acknowledged and addressed to reduce the likelihood that disparate care continues.

Findings from this study also highlight the need for professional development of staff related to assessment and management of complex conditions and equipment. NH residents with repeat transfers were largely impacted by multiple, complex medical and mental health conditions that require staff to have a specialized skillset. In all the MOQI homes, APRNs were available for nursing staff to discuss assessments and the need for hospital transfer and to assist with decision making on whether a transfer was needed. APRNs were rarely consulted in the cases of repeat transfers. Professional development for nursing staff may benefit from training on collaborative care and consultation when deliberating on decisions to transfer. In this study, residents and families often voiced preferences to have the resident transfer. Additional education and training for professional and other direct care staff on communicating with residents and their family advocates to understand their concerns and to share information about the scope of practice within NHs may also help families and residents better understand when transfer is clinically necessary.

### Limitations

There are limitations to consider as the results are interpreted. Although several NHs are included in the study, they are from one state in primarily one geographic region spanning urban and rural areas. These NHs participated in MOQI and had a full-time APRN embedded on-site; thus, broad generalizability of the results is limited. However, the NHs in this study faced many of the same challenges all NHs face with hospital transfers including clinically complex residents, residents and family influences, and organizational factors such as limited resources suggesting there is application to most US NHs. An additional limitation is the use of the INTERACT ACT which was intended for quality improvement purposes and not designed for research. Finally, as a retrospective analysis, the study team could not clarify APRN responses to the ACT tool questions therefore limiting our understanding of their responses. Interviews with APRNs as well as access to the residents’ NH and hospital medical records would have provided important clarification, for example why resident code status may have varied between transfers, why resident/family conversations about goals of care and advance care planning did/did not occur, or even why some residents were admitted to the hospital versus only being seen in the ED. However, written details about individual transfers, when provided, enhanced the quantitative findings, providing needed context to facilitate interpretation of the data. Additional study is needed to provide further understanding of these important issues.

## Conclusions

Repeat transfers reflected a mix between potentially avoidable and unavoidable transfers depending on resident acuity at the time of transfer and what clinical management, if any, was attempted prior to transfer. APRN responses about root causes of potentially avoidable transfers often indicated earlier communication between staff and/or with residents and family advocates as well as accessing available NH resources and staff training in complex care would have likely prevented the need for many of the repeat transfers.

The issue of repeat transfers from NH to hospital is more than the obvious problem of costs of care, but also quality of care and quality of life for NH residents. Experiencing a hospital transfer is difficult both physically and psychologically and often the resulting functional decline cannot be overcome, leaving residents more disabled. NH staff need to provide care to avoid the need for hospitalization, if possible. Staff also need to develop with residents/families realistic goals of care and plan for end-of-life care of their choice, including open discussions among family members, residents, and staff. Results of this analysis of repeat transfers can help provide insight and guide NH staff education to help them effectively provide care that prevents changes in health conditions and helps residents plan for best managing their health and end of life.

## Data Availability

The datasets used for this study are available from the corresponding author upon reasonable request.

## Abbreviations

MOQI: Missouri Quality Initiative
NH: Nursing home(s)
CMS: Centers for Medicare and Medicaid Services
APRN: Advanced practice registered nurse
